# Exploring inertial sensor-based balance biomarkers for early detection of mild cognitive impairment

**DOI:** 10.1038/s41598-024-59928-1

**Published:** 2024-04-29

**Authors:** Mobeena Jamshed, Ahsan Shahzad, Farhan Riaz, Kiseon Kim

**Affiliations:** 1 Department of Computer and Software Engineering, National University of Sciences and Technology, Islamabad, 44000 Pakistan; 2 School of Computer Science, University of Lincoln, Lincoln, LN67TS, UK; 3 School of Electrical Engineering and Computer Science, Gwangju Institute of Science and Technology, Gwangju, 61005 South Korea

**Keywords:** Machine learning, Data acquisition, Diagnostic markers

## Abstract

Dementia is characterized by a progressive loss of cognitive abilities, and diagnosing its early stages Mild Cognitive Impairment (MCI), is difficult since it is a transitory state that is different from total cognitive collapse. Recent clinical research studies have identified that balance impairments can be a significant indicator for predicting dementia in older adults. Accordingly, the current research focuses on finding innovative postural balance-based digital biomarkers by using wearable inertial sensors and pre-screening of MCI in home settings using machine learning techniques. For this research, sixty subjects (30 cognitively normal and 30 MCI) with waist-mounted inertial sensor performed balance tasks in four different standing postures: eyes-open, eyes-closed, right-leg-lift, and left-leg-lift. The significant balance biomarkers for MCI identification are discovered by our research, demonstrating specific characteristics in each of these four states. A robust feature selection approach is ensured by the multi-step methodology that combines the strengths of Filter techniques, Wrapper methods, and SHAP (Shapley Additive exPlanations) technique. The proposed balance biomarkers have the potential to detect MCI (with 75.8% accuracy), as evidenced by the results of machine learning algorithms for classification. This work adds to the growing body of literature targeted at enhancing understanding and proactive management of cognitive loss in older populations and lays the groundwork for future research efforts aimed at refining digital biomarkers, validating findings, and exploring longitudinal perspectives.

## Introduction

Neurodegenerative disorders, particularly dementia, provide a serious issue in modern healthcare because they cause a steady and long-term loss in cognitive abilities. This deterioration has a broader impact on language proficiency, memory retention, and executive capacities, while also causing motivational deficiencies, motor impairments, and emotional distress. The growth of these symptoms not only reduces the autonomy of people affected, but also has a significant impact on their overall well-being and that of their caretakers.

Mild Cognitive Impairment (MCI) develops as a critical point on Alzheimer’s Disease (AD) continuum in this complicated terrain of cognitive decline^1^. Each year, nearly 10% to 15% of the elderly people with MCI progress to dementia and 60–70% of dementia cases are caused by Alzheimer’s Disease (AD)^2^ . MCI is crucial in the early diagnosis and intervention efforts aimed at minimizing the impact of cognitive impairment because it serves as a vital connection between the pre-clinical phases of AD and the full-fledged onset of AD-related dementia.

Evaluation of motor function, including gait and balance, in the elderly population may be a helpful clinical tool for forecasting many clinical outcomes, including mortality, neurological illness^3^, cognitive impairment^4^ and fall risk^5^. Postural control, or balance, is the result of the cooperative efforts of several body systems, including the vestibular, motor, cognitive, visual, and sensory. As a result, abnormalities in any of these systems, including neuropathology and cognitive decline, can cause deficits in balance^3^. The presence or the severity of gait and/or balance disturbances are associated with a higher risk of Alzheimer’s dementia^6^. According to research^7^, there were notable variations in the MCI groups’ static balance performance when contrasted with the typical aging group. As a result, standing balance may be a helpful biomarker for the development of neurodegenerative diseases and mild cognitive impairments.

Accelerometers and gyroscopes are examples of wearable, wireless technology that has recently come to light as a viable alternative for clinical and laboratory testing^8,9^. Inertial sensors are often integrated into wearable devices, enabling real-time capture of movement and acceleration data for analyzing posture and balance. Clinical tests can be conducted in conjunction with these tools, giving useful data that will help make better-informed decisions about extent of cognitive decline and the treatments that will follow^4,10^.

Research on the use of static balance biomarkers derived from wearable inertial sensors to distinguish MCI patients from cognitively normal individuals is lacking. In studies^11,12^, correlation between cognitive dysfunction and standing postural balance have been reported in MCI patients using force platforms for assessing static balance parameters. A recent study^13^, developed new balance stability indicator with area under the curve (AUC=0.806), using stabilometer, whereas researchers in^14^ assessed and compared the static balance ability of the older adults with MCI standing on soft and hard support surfaces.

Some research is being done on using dynamic balancing tests which involves moderate activity, such as walking, which are not very easy to perform in older patients^8,15^. Static standing balance measurement may be a simple method that requires less physical load in older adults^16^. Furthermore, selection of feature set is very important for improving the accuracy of classification when machine learning techniques are being employed^17^.

Therefore, we aimed to identify key balance biomarkers of MCI using wearable inertial sensor signals for early diagnosis using machine learning techniques. The contributions of our paper are summarized as follows:

- To extract and evaluate a set of objective measures of balance dysfunction that are different in individuals with MCI and healthy controls using wearable inertial sensors involving static balance metrics.
- Our study examines changes in static postural sway in four distinct scenarios, i.e., Eyes Open, Eyes Closed, Right Leg Lift, and Left Leg Lift, to see which one yields the greatest outcomes.
- To analyze the performance of key balance features for early detection of MCI using various machine learning models.

## Methods

### Participants

At National Research Center for Dementia, Gwangju, South Korea, selection of 60 participants was carefully carried out to remove demographics biases such as age, height, weight etc. They were divided into two groups: 30 participants classified as Cognitively Normal (CN) and another 30 diagnosed with Mild Cognitive Impairment (MCI).

All the subjects were evaluated and diagnosed based on assessments conducted by medical professionals at Chosun University Hospital and Chonnam National University Hospital in Gwangju. The Gwangju Institute of Science and Technology’s (GIST) Institutional Review Board approved the study protocols and all experimental procedures were carried out according to the approved guidelines and regulations. Before the trials began, all subjects and/or their guardians gave written, informed consent.

Each subject underwent a comprehensive set of tests, including MRI scans to investigate brain anatomy and PET scans to detect Beta-amyloid plaques. To evaluate cognitive ability, the Mini-Mental State Examination (MMSE)^18^ was utilized, with MCI diagnosis determined by scores greater than 1.5 standard deviations^19,20^. The neuro-psychological assessments were conducted using Seoul Neuro-psychological Screening Battery (SNSB)^21^ consisting of five major cognitive domains: attention, language, visuo-spatial, memory, and frontal/executive domains. Participants with focal brain lesions, dementia unrelated to Alzheimer’s disease, and other severe medical, neurological, or mental problems that could impair cognitive functions and balance were specifically excluded from the study.

Analysis of Variance (ANOVA) was utilized to compare the two groups’ demographic and cognitive characteristics. The demographic and neuropsychological outcomes for all participants, along with corresponding p-values, are shown in Table 1. Importantly, no noteworthy differences in age, gender, height, weight, or education level were discerned between the Cognitively Normal (CN) and Mild Cognitive Impairment (MCI) groups.

**Table 1:** Demographics of subjects.

Demographics	CN (Mean ± Std)	MCI (Mean ± Std)	p-value
Subjects	30	30	–
MMSE	27.53 ± 2.029	25.87 ± 3.36	0.02357
Age (yrs)	74.77 ± 4.797	76.53 ± 3.45	0.10696
Gender (M/F)	16/14	20/10	0.29985
Height(cm)	160.35 ± 7.06	162.76 ± 8.72	0.24279
Weight (kg)	61.64 ± 7.21	63.26 ± 8.24	0.42204
Education (yr)	9.97 ± 4.498	10.70 ± 4.55	0.53263

### Data Acquisition Protocol

**Figure 1:**
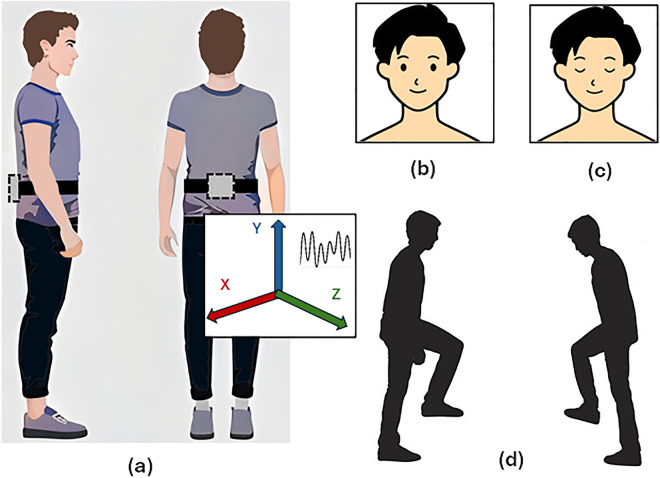
(**a**) Sensor mounted on participant’s back, with data collection in 4 conditions, (**b**) Eyes Open (EO), (**c**) Eyes-Closed (EC), (**d**) Right-Leg (RL) and Left-Leg Lift (LL).

The experimental protocol involved subjects wearing the Shimmer 3 inertial sensor^22^, a wearable device with a tri-axial accelerometer and tri-axial gyroscope. This sensor was securely positioned on the lower back of participants (specifically, the L3-L5 vertebrae) using an adjustable belt and was closely monitored by an observer. The location selected is close to the Center-of-Mass (COM) of the human body, and whole-body movements are revealed using acceleration data from a sensor positioned close to COM^23^. Under four different conditions, subjects were instructed to remain in an upright posture with their arms by their sides: eyes-open (EO), eyes-closed (EC), right-leg lift (RL), and left-leg lift (LL), as shown in Fig. 1. The data was collected from two trials of each subject. Prior to data collection, the sensor underwent pre-calibration following the outlined procedure in^24^, ensuring accuracy. It was configured to measure within a range of ± 4 g, and the sampling rate was set at 64 Hz. Bluetooth was used to transfer data to a nearby laptop, and ConsensysPRO^25^ was used to synchronize the data in real time.

During signal processing, the collected data underwent filtration through an 8th-order zero-phase low-pass Butterworth filter. The 5 Hz cutoff frequency was chosen for the filter, contributing to the refinement of the sensor data for subsequent analyses. The sensor’s x-, y-, and z-axes corresponded to the Medio-Lateral (ML), Vertical (V) and Antero-posterior (AP) orientations of the participants, respectively.

### Feature Extraction

We systematically assessed a considerable number of features from literature across diverse domains related to balance and falls risk in aging and neuro-degenerative diseases. The focus of our study is on computing several standard measures within the quantitative balance parameters, specifically in the time and frequency/spectral domains, to quantify postural balance. A comprehensive set of 76 postural sway measures was employed, comprising 43 features associated with time and 33 features linked to frequency/spectral characteristics, as shown in Table 2. Every feature was computed for all of the four standing balance conditions for every subject.

**Table 2:** Summary and brief description of the features.

Feature no.	Feature name	Domain	Unit	Definition
0–3	Mean Distance (MDIST)^26^	Time	$m/s^2$	Mean distance from the center of acceleration trajectory
4	Average Absolute Acceleration Magnitude Variation (AAMV)^27,28^	Time	m $/s^2$	Variation in average acceleration magnitude from mean
5–8	Path length (TOTEX)^26^	Time	$m/s^2$	Total length of acceleration path approximated by sum of the distances between consecutive points
9–12	Normalized Path length (NPL)^29^	Time	$m/s^2$	Total length of acceleration path normalized
13–16	Mean Sway Velocity (MVELO)^26^	Time	$m/s^2$	Average Velocity
17–20	Range of Acceleration (R)^26,30^	Time	$m/s^2$	Range of acceleration signals
21–24	Root Mean Square of Acceleration (RMS)^5,30–32^	Time	$m/s^2$	RMS of the acceleration
25–28	Root Mean Square of Angular Velocity (RMS-G)^5^	Time	*deg*/*s*	RMS of the angular velocity
29	95% Confidence Circle Sway Area (AREA-CC)^3,5,26^	Time	$m^2/s^4$	Approx 95% of the distances from the mean COM
30	95% Confidence Ellipse Area (AREA-CE)^5,26,31^	Time	$m^2/s^4$	Area of the 95% bivariate confidence ellipse
31	Sway Area (AREA-SW)^5,26,31^	Time	$mm^2/s^5$	Area enclosed by COM path per unit of time
32–35	Jerk^3,5,31^	Time	$m^2/s^5$	Area enclosed by COM path per unit of time
36–38	Total Spectral Power (TP)^26,30^	Frequency	$\upmu$	Total power of the spectrum of accelerations
39–41	Spectral Edge Frequency (SEF)^5^	Frequency	Hz	Frequency containing 95% of the total power
42–44	Spectral Edge Frequency-Angular Velocity (SEF-G)^5^	Frequency	Hz	Frequency containing 95% of the total power of Angular Velocity
45–47	Median Frequency (Med)^5,30,33^	Frequency	Hz	Frequency containing 50% of the total power
48–50	Peak Frequency	Frequency	Hz	Frequency with the highest value
51–53	Centroidal Frequency (C-FREQ)^26,30^	Frequency	Hz	Frequency at which spectral mass is concentrated
54–56	Frequency Dispersion (FREQ-D)^26,30^	Frequency	–	Measure of the variability of the frequency content of the power spectral density
57–60	Spectral Entropy (Spec-Entropy)^33^	Frequency	–	Power spectrum entropy of accelerations
61–64	Spectral Entropy-Angular Velocity (Spec-Entropy-G)^33^	Frequency	–	Power Spectrum entropy of Angular Velocity
65–67	Summed Axis Acceleration (SAA)^5^	Time	$m/s^2$	Sum of all samples of individual acceleration signals
69	Summed Magnitude Area (SMA)^5^	Time	$m/s^2$	Sum of absolute of all accelerations signals
69–71	R-SAA-SMA^5^	Time	$m/s^2$	Ratio of SAA to SMA
72–75	Mean Frequency (MFREQ)^5,33^	Frequency	Hz	Mean frequency of the acceleration power spectrum

Equation (1) is used to calculate acceleration Signal Vector Magnitude (SVM). Some parameters (36–56, 65–67, 69–71) are computed for each axis: ML, V, AP; other parameters (0–3, 5–16, 21–28, 57–64, 72–75) are computed for SVM as well as for each axis: ML, V, AP; few parameters (17–20, 32–35) are calculated for some of these planes: AP-ML, ML-V, AP-V, and rest of the parameters (4, 29–31, 68) are calculated just for SVM.

1 $$\begin{aligned} SVM (n) = \sqrt{A_x (n)^2 + A_y (n)^2 + A_z (n)^2} \end{aligned}$$

### Feature Selection

Feature ranking was executed through the utilization of a “Leave-One-Subject-Out (LOSO)” cross-validation technique for each session. The dataset was divided iteratively into training and test sets during this process, with one subject removed at a time. Two distinct categories of feature-ranking techniques were applied. Firstly, Filter Methods were employed, including ANOVA (Analysis of Variance) and Mutual Information, which assess the relevance of features independently of the classification model. Mutual Information (MI) measures the extent to which knowledge of one quantity reduces uncertainty about another, while ANOVA compares the variation in the group means. Secondly, Wrapper Methods: Random Forest, Support Vector Machine (SVM) were utilized to evaluate feature subsets by considering how they affect the performance of a specific classification model.

The value of each feature was calculated by the number of times it was featured in the top 15 feature list throughout all 60 folds. Following that, a score was assigned to each feature, offering insight into the consistent significance of features across diverse subject exclusions. Finding strong features that were consistently significant over a range of subjects and folds was the aim of this technique, which laid the groundwork for further analysis processes. The scores from the feature-ranking techniques were combined to determine the final score, which indicates the relative weight of feature across the various approaches.

In a research work^34^, hybrid feature selection approach is proposed, by combining filter and wrapper methods . In their study, the features were first ranked based on the ranking criteria’s and then a wrapper algorithm is invoked to generate a subset from the ranked features. However, our study used both filter and wrapper methods for assigning score to the feature.

**Figure 2:**
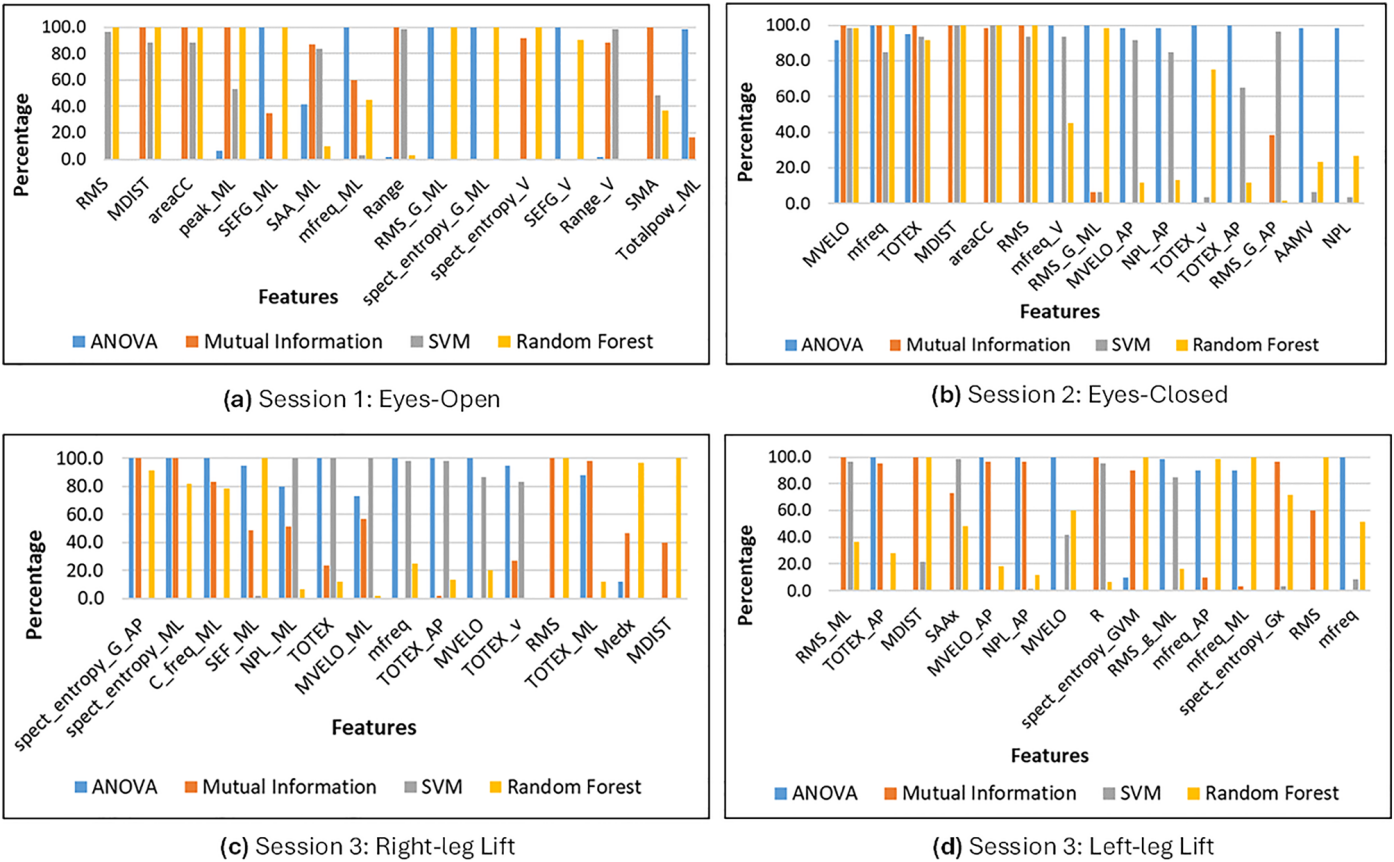
Results of each ranking technique for top 15 features.

The key features identified for each session are shown in Fig. 2 and Supplementary Table S1. This extraction process ensured that the most important features would be selected by employing multiple feature-ranking techniques. Table 3 presents the top 10 salient features found in each session.

**Table 3:** Key features identified in each session.

Session-1: Eyes Open	Session-2: Eyes Closed	Session-3: Right Leg Lift	Session-4: Left Leg Lift
RMS	MVELO	Spect-Entropy-G-AP	RMS-ML
MDIST	Mean-freq	Spect-Entropy-ML	TOTEX-AP
Area-CC	TOTEX	C-freq-ML	MDIST
Peak-ML	MDIST	SEF-ML	SAA-ML
SEFG-ML	Area-CC	NPL-ML	MVELO-AP
SAA-ML	RMS	TOTEX	NPL-AP
Mean-freq-ML	Mean-Freq-V	MVELOL-ML	MVELO
Range	RMS-G-ML	Mean-Freq	Range
RMS-G-ML	MVELO-AP	TOTEX-AP	Spect-Entropy-G-V
Spect-Entropy-G-ML	NPL-AP	MVELO	RMS-G-ML

## Results

### Model Evaluation

**Figure 3:**
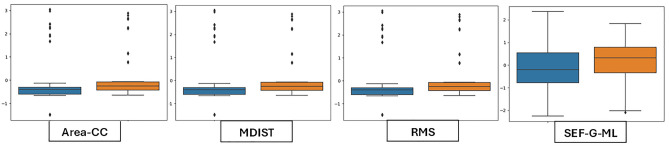
Session-1 (Eyes-Open)—Illustration of balance data across MCI and Controls. Orange indicates CN and blue represents MCI subject groups.

**Figure 4:**
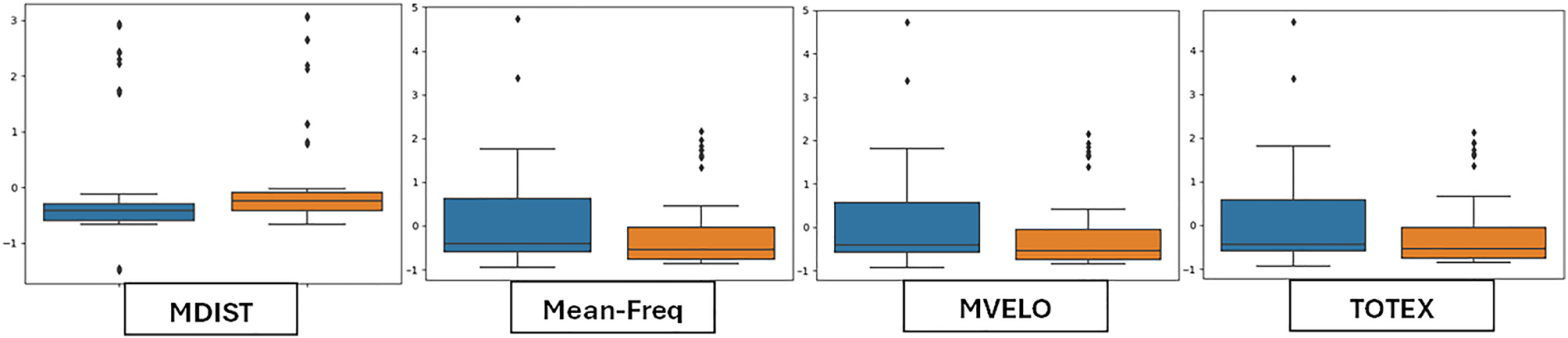
Session-2 (Eyes-Closed)—Illustration of balance data across MCI and Controls. Orange indicates CN and blue represents MCI subject groups.

**Figure 5:**
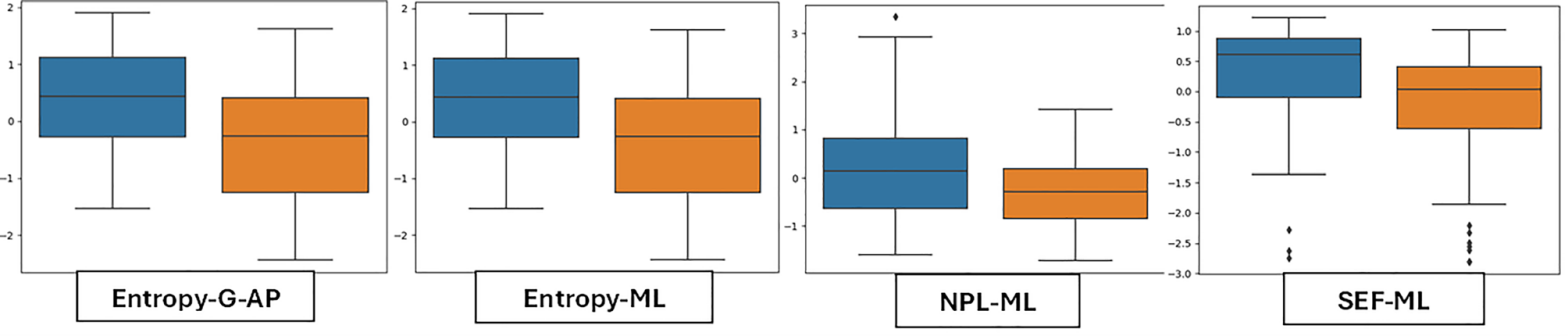
Session-3 (Right-Leg-Lift)—Illustration of balance data across MCI and Controls. Orange indicates CN and blue represents MCI subject groups.

**Figure 6:**
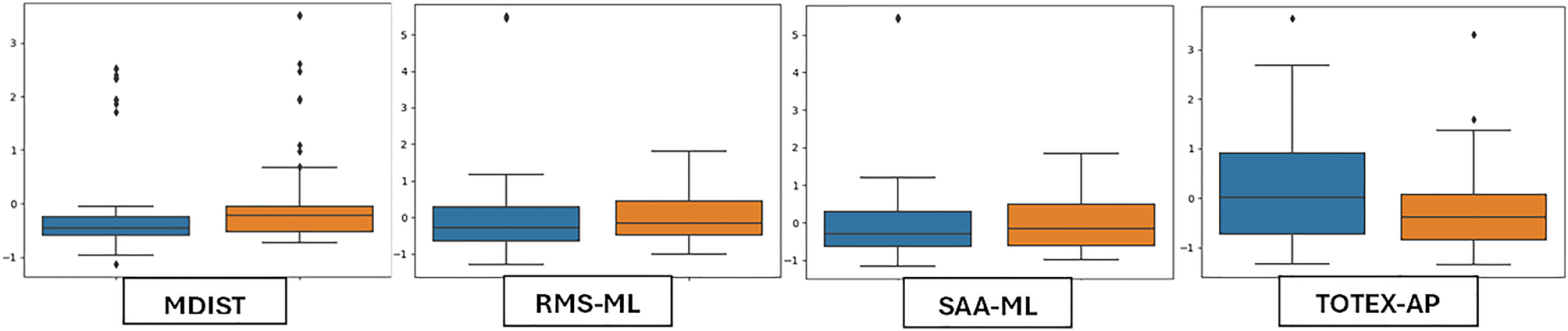
Session-4 (Left-Leg-Lift)—Illustration of balance data across MCI and Controls. Orange indicates CN and blue represents MCI subject groups.

To analyze the variability of features, box plots are employed to display the distributional characteristics of the data. In Session-1, RMS, SEF-G-ML and Area-CC have lower values for MCI than CN (Fig. 3). MDIST exhibits lower values for MCI than CN in all conditions. The MCI group had considerably higher values than the CN group for MVELO, M-freq and TOTEX (Fig. 4). Similarly, Spect-Entropy-G-AP, Spect-Entropy-ML, SEF-ML and TOTEX-AP have higher values for MCI than CN, hence contributing to discriminating the classification groups (Figs. 5, 6).

### Explainability using SHAP

**Figure 7:**
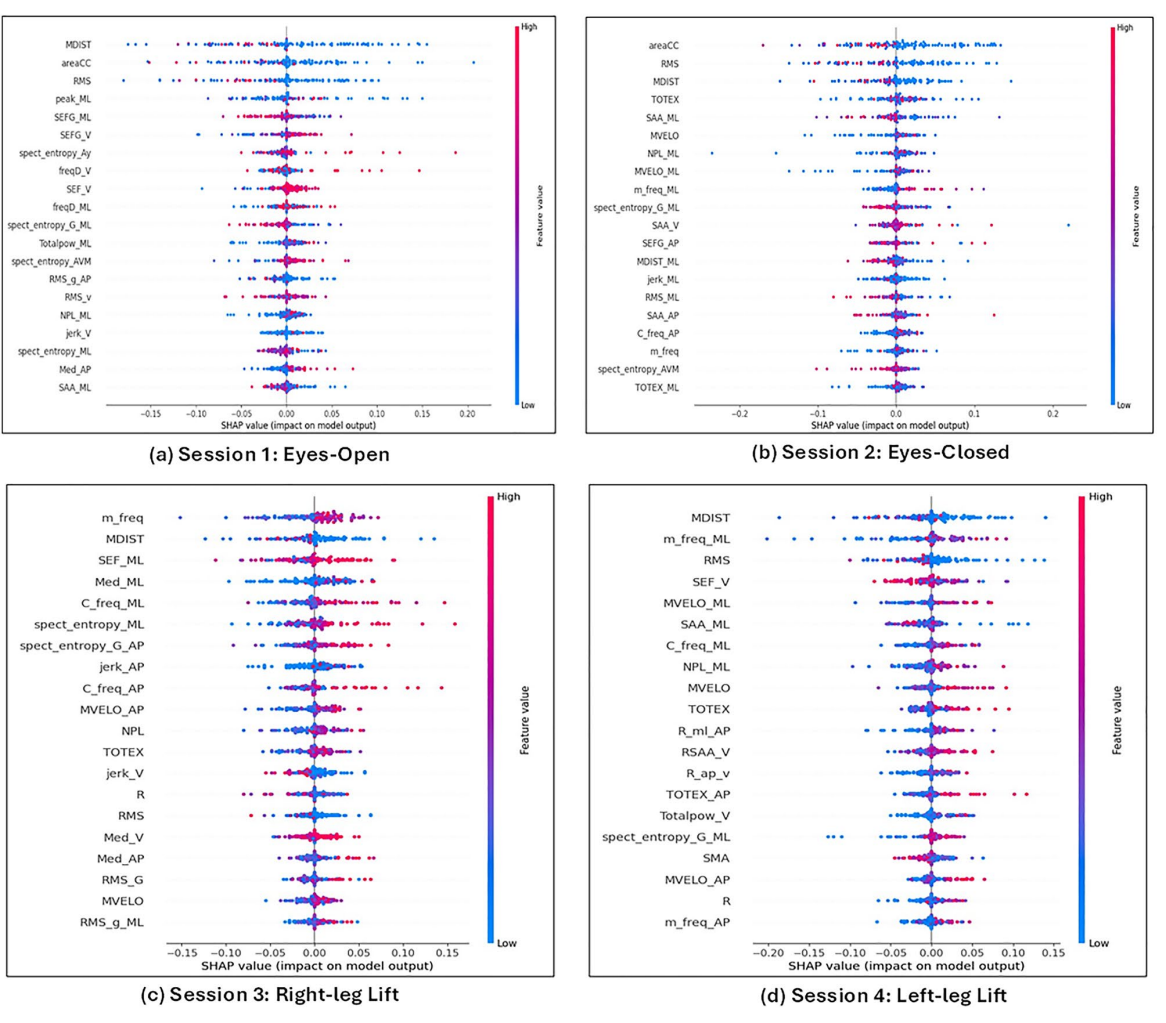
SHAP summary plots showing the relationship between the features’ SHAP values and their impact.

The feature set was further analyzed by SHAP (Shapley Additive exPlanations), suggested by Lundberg and Lee^35^. Game theory forms the basis of SHAP^36^, and it provides a way to calculate each feature’s contribution to the model. The greater contribution a feature makes to the model’s prediction, the more significant it is. A concise description of the impact distribution of features on Random Forest model and the relationship between those features’ SHAP values and their impact, is given by the SHAP summary charts in Fig. 7. After comparing the important features based on SHAP impact value (Fig. 7) and our top features (Table 3), it is important to note that all key features identified by our research were included in the list of SHAP, hence proving the effectiveness of our method in identifying important biomarkers for early detection of MCI. In Session-1 (EO), maximum similarity is obtained, followed by Session-2. This also explains the highest accuracy results obtained in Session-1 (Table 4). However, in Session-3 and Session-4, similarity between identified features extends to the top 10 or 15 features. Therefore, we have observed that a larger set of features are required to differentiate between controls and MCI in those conditions, as frequency/spatial features are also involved in that scenario.

**Figure 8:**
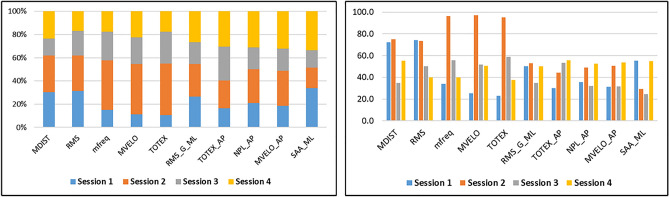
Top key Features identified across all sessions. Contribution of scores from each of the four sessions are shown in two different perspectives.

Additional investigation was done to find significant features across all sessions. To achieve that, scores of top features from all sessions were combined. The top 10 features that were determined to be the most significant throughout all sessions are displayed in Fig. 8. The final score for each feature is calculated from the contribution from each of the four sessions, as given in Supplementary Table S2.

### Classification results

The top 15 features in each session were then used as the foundation for the classification stage, where Support Vector Machine (SVM)^37^, Random Forest^38^, and Ensemble Models (comprising Majority Voting and Gradient Boosting^39^) were employed. Leave-One-Subject-Out (LOSO) cross-validation strategy was applied to assess the effectiveness of the chosen characteristics for MCI and CN classification. This required categorizing the data, systematically eliminating subjects one at a time, and obtaining the predictions across all 60 folds. Accuracy, precision, specificity, and sensitivity measures were calculated based on the predicted values, providing a thorough assessment of the classification models’ performance. This phase demonstrated a direct correlation between feature selection and prediction accuracy, providing valuable insights into the features’ potential utility as balance biomarkers for MCI early identification. The best results obtained by using top features for classification are summarized in Table 4.

MMSE is frequently administered in clinical contexts to evaluate cognitive impairment. MMSE achieved Area-under-the-curve (AUC) score AUC=0.669, on our dataset. Our findings demonstrate that top significant features outperformed MMSE in terms of performance, Table 4.

**Table 4:** Summarized classification results of various machine learning models.

Classification Model	Accuracy (%)	Precision (%)	Sensitivity (%)	Specificity (%)
*Session 1: Eyes Open*				
SVM	${\textbf {75.8}}$	80.4	68.3	83.3
Random Forest	68.3	69.6	65	71.7
Ensemble (Majority Voting)	72.5	76.5	65	80
*Session 2: Eyes Closed*				
SVM	60.8	62.3	55	66.7
Random Forest	65.8	66.7	63.3	68.3
Ensemble (Majority Voting)	62.5	66.7	50	75
*Session 3: Right Leg Lift*				
SVM	62.5	62.7	61.7	63.3
Random Forest	69.2	69.5	68.3	70
Ensemble (Majority Voting)	64.2	67.4	55	73.3
*Session 4: Left Leg Lift*				
SVM	66.7	67.2	65	68.3
Random Forest	67.5	69.8	61.7	73.3
Ensemble (Majority Voting)	66.7	66.7	68	68.1

## Discussion and conclusion

The study used wearable inertial sensors to analyze various static balance metrics in eyes-open, eyes-closed, right-leg lift, and left-lift scenarios. Our research pioneers the use of wearable sensor data for classifying MCI patients and individuals without cognitive impairment through static balance biomarkers. Existing research has mainly focused on finding potential biomarkers and analyzing them statistically^3,7,10^ or by finding correlation with MMSE^16^. However, we have employed a multi-step methodology based on machine learning techniques for selecting features, combining the strengths of filter and wrapper methods^34^, and subsequently employing classification algorithms. Boxplots and SHAP have also been utilized for statistical analysis of key features.

Under several static balance settings, we have discovered and analyzed features that significantly differ in MCI patients. Root Mean Square (RMS), mean distance (MDIST), 95% Confidence Circle Sway Area (AREA-CC), Path Length (TOTEX), and Mean Sway Velocity (MVELO) are the principal features of relevance in the Time domain, Table 3 and Fig. 8. Substantial biomarkers for balance in the frequency or spectrum domain are Spectral Edge frequency (SEF), Mean frequency (M-Freq), Peak frequency (P-Freq), Centroid frequency (C-Freq), and Entropy (Spect-Entropy), Table 3 and Fig. 8.

Previous findings have also identified RMS of the acceleration signal^9,31^, Mean Frequency^17^ and 95% AREA-CE^14^ as important biomarkers for static balance impairment. Our results are consistent with prior research^7^, which states that in the setting of eyes-open conditions, balancing metrics such as anterior-posterior (AP) sway and medio-lateral (ML) sway position were found to be relevant discriminators, but not in eyes-closed conditions, as the defective central processing of visual information is linked to balance abnormalities associated with MCI. One possible explanation for this is that MCI slows down information processing, and these groups rely on visual cues to maintain postural stability.

The key features identified in feature selection process were provided as input to different classification models: Support Vector Machine (SVM), Random Forest, and Ensemble Model (Majority Voting). The results (Table 4) show that best classification results are obtained using SVM yielding 75.8% accuracy in eyes-open condition. However, Random Forest Classifier can be regarded as the best performing model across all sessions, by obtaining more than 65% accuracy in four different conditions. Static eyes-open balance features were discovered to have promising routes for early identification as they stood out as particularly unique, Table 4 and Fig. 7.

Although our study adds to the expanding corpus of research on dementia, it is critical to recognize its limitations. To achieve a thorough understanding of MCI, more research is necessary to evaluate the identified biomarkers in longitudinal cross-cultural settings as well as investigate the integration of other modalities. Furthermore, longitudinal studies are essential for monitoring the evolution of biomarkers related to balance over time.

Our study shows that wearable inertial sensors can be a viable tool for early dementia identification, and it also emphasizes the significance of postural balance measurement in MCI detection. Using these methods could make it possible to identify dementia and Alzheimer’s disease early on even in home settings. Furthermore, the study provides strong data outlining the critical parameters for assessing balance and lays the groundwork for future research aimed at improving, validating, and optimizing a standardized clinical motor assessment technique customized for people with MCI.

### Supplementary Information

Supplementary Information.

## Data Availability

Raw data was generated at the National Research Center for Dementia, Gwangju, South Korea.
