# Supplementary material for: Exploring inertial sensor-based balance biomarkers for early detection of mild cognitive impairment
Source: Sci Rep. 2024 Apr 29;14:9829. doi: 10.1038/s41598-024-59928-1 (PMC11059265; doi:10.1038/s41598-024-59928-1)
Supplement: Supplementary file 1 — Supplementary Information. [file 41598_2024_59928_MOESM1_ESM.pdf]

Supplementary Data

Exploring Inertial Sensor-based Balance Biomarkers for Early Detection of Mild Cognitive Impairment

Mobeena Jamshed[1, +] , Ahsan Shahzad [*,1,+], Farhan Riaz [2], and Kiseon Kim [3]

[1] Department of Computer and Software Engineering, National University of Sciences and Technology, Islamabad, 44000, Pakistan
[2] School of Computer Science, University of Lincoln, United Kingdom
[3] School of Electrical Engineering and Computer Science, Gwangju Institute of Science and Technology, Gwangju, 61005, South Korea
* ahsan.shahzad@ceme.nust.edu.pk
+ these authors contributed equally to this work.

Session-1: Eyes Open							
Rank	Feature No	Feature Name	Filter Methods		Wrapper Methods		Total Score (%)
			ANOVA	Mutual Information	Support Vector Machine	Random Forest	
1	21	**RMS**	0.0	100.0	96.7	100.0	**74.2**
2	0	**MDIST**	0.0	100.0	88.3	100.0	**72.1**
3	29	**areaCC**	0.0	100.0	88.3	100.0	**72.1**
4	48	**peak_ML**	6.7	100.0	53.3	100.0	**65.0**
5	42	**SEFG_ML**	100.0	35.0	0.0	100.0	**58.8**
6	65	**SAA_ML**	41.7	86.7	83.3	10.0	**55.4**
7	73	**mean_freq_ML**	100.0	60.0	3.3	45.0	**52.1**
8	17	**Range**	1.7	100.0	98.3	3.3	**50.8**
9	26	**RMS_G_ML**	100.0	0.0	0.0	100.0	**50.0**
10	62	**spect_entropy_G_ML**	100.0	0.0	0.0	100.0	**50.0**
11	60	**spect_entropy_A_V**	0.0	91.7	0.0	100.0	**47.9**
12	43	**SEFG_V**	100.0	0.0	0.0	90.0	**47.5**
13	20	**Range_v**	1.7	88.3	98.3	0.0	**47.1**
14	68	**SMA**	0.0	100.0	48.3	36.7	**46.3**
15	36	**Totalpow_ML**	98.3	16.7	0.0	66.7	**45.4**

(A): Session-1: Eyes Open

Session-2: Eyes Closed							
Rank	Feature No	Feature Name	Filter Methods		Wrapper Methods		Total Score (%)
			ANOVA	Mutual Information	Support Vector Machine	Random Forest	
1	13	**MVELO**	91.67	100.00	98.33	98.33	**97.08**
2	72	**mean_freq**	100.00	100.00	85.00	100.00	**96.25**
3	5	**TOTEX**	95.00	100.00	93.33	91.67	**95.00**
4	0	**MDIST**	0.00	100.00	100.00	100.00	**75.00**
5	29	**areaCC**	0.00	98.33	100.00	100.00	**74.58**
6	21	**RMS**	0.00	100.00	93.33	100.00	**73.33**
7	74	**mfreq_V**	100.00	0.00	93.33	45.00	**59.58**
8	26	**RMS_G_ML**	100.00	6.67	6.67	98.33	**52.92**
9	16	**MVELO_AP**	98.33	0.00	91.67	11.67	**50.42**
10	12	**NPL_AP**	98.33	0.00	85.00	13.33	**49.17**
11	7	**TOTEX_v**	100.00	0.00	3.33	75.00	**44.58**
12	8	**TOTEX_AP**	100.00	0.00	65.00	11.67	**44.17**
13	28	**RMS_g_AP**	0.00	38.33	96.67	1.67	**34.17**
14	4	**AAMV**	98.33	0.00	6.67	23.33	**32.08**
15	9	**NPL**	98.33	0.00	3.33	26.67	**32.08**

(B): Session-2: Eyes Closed

Session-3: Right Leg Lift							
Rank	Feature No	Feature Name	Filter Methods		Wrapper Methods		Total Score (%)
			ANOVA	Mutual Information	Support Vector Machine	Random Forest	
1	64	**spect_entropy_G_AP**	100.00	100.00	0.00	91.67	**72.92**
2	59	**spect_entropy_ML**	100.00	100.00	0.00	81.67	**70.42**
3	51	**C_freq_ML**	100.00	83.33	0.00	78.33	**65.42**
4	39	**SEF_ML**	95.00	48.33	1.67	100.00	**61.25**
5	10	**NPL_ML**	80.00	51.67	100.00	6.67	**59.58**
6	5	**TOTEX**	100.00	23.33	100.00	11.67	**58.75**
7	14	**MVELO_ML**	73.33	56.67	100.00	1.67	**57.92**
8	72	**mfreq**	100.00	0.00	98.33	25.00	**55.83**
9	8	**TOTEX_AP**	100.00	1.67	98.33	13.33	**53.33**
10	13	**MVELO**	100.00	0.00	86.67	20.00	**51.67**
11	7	**TOTEX_v**	95.00	26.67	83.33	0.00	**51.25**
12	21	**RMS**	0.00	100.00	0.00	100.00	**50.00**
13	6	**TOTEX_ML**	88.33	98.33	0.00	11.67	**49.58**
14	45	**Medx**	11.67	46.67	0.00	96.67	**38.75**
15	0	**MDIST**	0.00	40.00	0.00	100.00	**35.00**

(C): Session-3: Right leg Lift

Session-4: Left Leg Lift							
Rank	Feature No	Feature Name	Filter Methods		Wrapper Methods		Total Score (%)
			ANOVA	Mutual Information	Support Vector Machine	Random Forest	
1	22	**RMS_ML**	0.00	100.00	96.67	36.67	**58.33**
2	8	**TOTEX_AP**	100.00	95.00	0.00	28.33	**55.83**
3	0	**MDIST**	0.00	100.00	21.67	100.00	**55.42**
4	65	**SAA_ML**	0.00	73.33	98.33	48.33	**55.00**
5	16	**MVELO_AP**	100.00	96.67	0.00	18.33	**53.75**
6	12	**NPL_AP**	100.00	96.67	1.67	11.67	**52.50**
7	13	**MVELO**	100.00	0.00	41.67	60.00	**50.42**
8	17	**Range**	0.00	100.00	95.00	6.67	**50.42**
9	58	**spect_entropy_G**	10.00	90.00	0.00	100.00	**50.00**
10	26	**RMS_G_ML**	98.33	0.00	85.00	16.67	**50.00**
11	75	**mean_freq_AP**	90.00	10.00	0.00	98.33	**49.58**
12	73	**mean_freq_ML**	90.00	3.33	0.00	100.00	**48.33**
13	62	**spect_entropy_G_ML**	0.00	96.67	3.33	71.67	**42.92**
14	21	**RMS**	0.00	60.00	0.00	100.00	**40.00**
15	72	**mean_freq**	100.00	0.00	8.33	51.67	**40.00**

(D): Session-4: Left Leg Lift

Table S1: Top 15 key features identified by combining scores from four feature-ranking techniques, ANOVA, Mutual Information, SVM and Random Forest

(A): Session-1: Eyes-Open, (**B):** Session-2: Eyes-Closed, (**C):** Session-3: Right-Leg-Lift, (**D):** Session-4: Left-Leg-Lift

Rank	Feature No	Feature Name	Session-1	Session-2	Session-3	Session-4	Total Score (%)
1	0	MDIST	72.08	75.00	35.00	55.42	59.38
2	21	RMS	74.17	73.33	50.00	40.00	59.38
3	72	mean_freq	34.17	96.25	55.83	40.00	56.56
4	13	MVELO	25.42	97.08	51.67	50.42	56.15
5	5	TOTEX	22.92	95.00	58.75	37.50	53.54
6	26	RMS_G_ML	50.00	52.92	35.00	50.00	46.98
7	8	TOTEX_AP	30.00	44.17	53.33	55.83	45.83
8	12	NPL_AP	35.42	49.17	32.08	52.50	42.29
9	16	MVELO_AP	31.25	50.42	31.67	53.75	41.77
10	65	SAA_ML	55.42	29.17	24.58	55.00	41.04

Table S2: Top 10 key features identified by combining scores from four different standing positions, Eyes-Open, Eyes-Closed, Right-Leg-Lift, Left-Leg-Lift

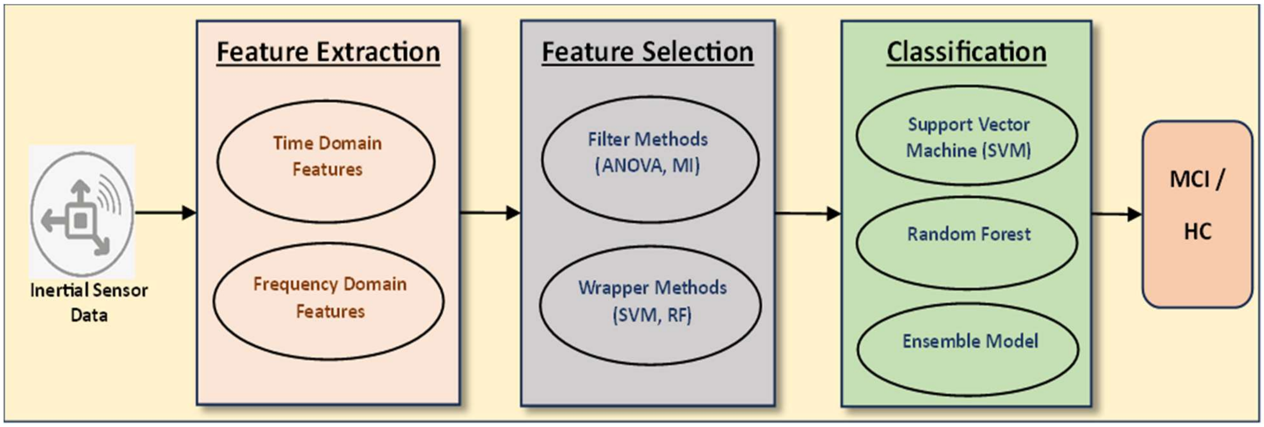

Figure S3: Flow diagram of proposed methodology.
